# Influence of the *cis*/*trans* configuration on the supramolecular aggregation of aryltriazoles

**DOI:** 10.3762/bjoc.15.282

**Published:** 2019-11-28

**Authors:** Sara Tejera, Giada Caniglia, Rosa L Dorta, Andrea Favero, Javier González-Platas, Jesús T Vázquez

**Affiliations:** 1 Instituto Universitario de Bio–Orgánica “Antonio González”, Departamento de Química Orgánica, Universidad de La Laguna, 38206 La Laguna, Tenerife, Spain

**Keywords:** circular dichroism, *cis*/*trans* configuration, gels, triazole, X-ray diffraction

## Abstract

The ability of *trans*- and *cis*-1,2-glucopyranosyl and cyclohexyl ditriazoles, synthesized by CuAAC "click" chemistry, to form gels was studied, their physical properties determined, and the self-aggregation behavior investigated by SEM, X-ray, and EDC studies. The results revealed that self-assembly was driven mainly by π–π stacking interactions, in addition to hydrogen bonding, with the aromatic rings adopting a high degree of parallelism, as seen in crystal packings and ECD data. Furthermore, π–bromine interactions between the bromine atom of the aryl substituents and the triazole units might also contribute to an overall stabilization of the supramolecular aggregation of bis(4-bromophenyl)triazoles. The *trans* or *cis* spatial disposition of the triazole rings is highly important for gelation, with the *cis* configuration having higher propensity.

## Introduction

Structures self-assembled by noncovalent interactions give rise to supramolecular architectures with specific physical and/or chemical properties. Gels are colloid systems in which the dispersed phase has combined with the dispersion medium to yield a semisolid material. Gels from low-molecular-weight gelators have potential applications in high-tech materials [1–3] and biomedical sciences [3–6]. Triazole derivatives have shown excellent gelation properties [7–10], in addition to a broad range of biological activities [11–14].

During the synthesis and characterization process of glucosyl mono- and ditriazole derivatives, which we carried out in order to analyze the use of aryltriazoles for the determination of absolute configuration [15], we serendipitously discovered that these glucosyl ditriazoles led to gels. Therefore, we carried out the corresponding supramolecular studies and report herein the ability of ditriazoles to form gels, their physical properties, as well as the dependence of these properties on the *cis*/*trans* relative configuration.

## Results and Discussion

**Scheme 1:**
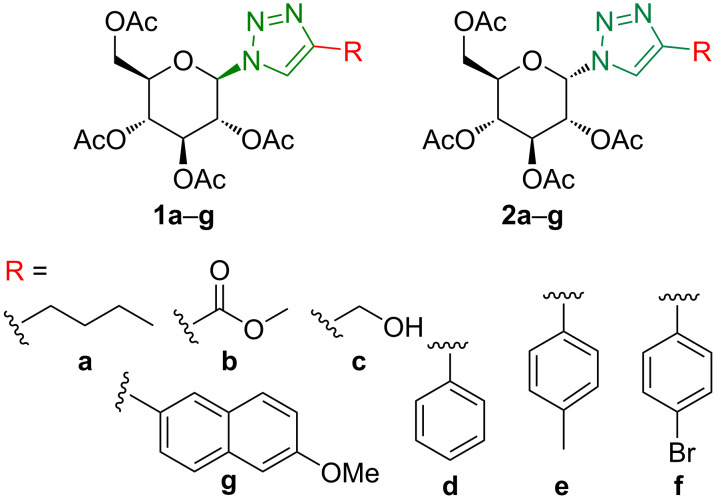
Structures of 4-substituted 1-glucopyranosyltriazoles **1a**–**g** and **2a**–**g** [15].

**Scheme 2:**
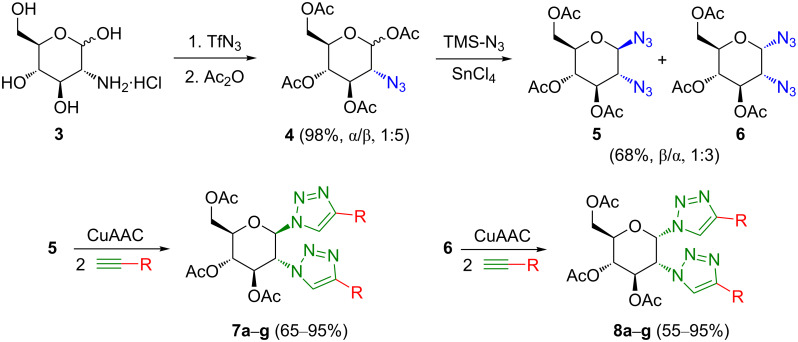
Synthesis of 1,2-*cis*-/*trans*-bistriazoles **7a**–**7g** and **8a**–**8g** [15].

A large set of mono- and ditriazoles was synthesized using cycloaddition reactions based on "click" chemistry [16–18] of azides and alkynes catalyzed by Cu(I) salts, the CuAAC reaction. Self-assembling properties were not observed for any of the prepared monotriazoles, namely the 4-substituted 1-glucopyranosyltriazoles **1a**–**g** and **2a**–**g** (Scheme 1) [15]. However, most ditriazoles **7a**–**g** and **8a**–**g** (Scheme 2) showed supramolecular features, i.e., their DMSO solutions prepared for NMR analyses spontaneously transformed into gels inside the NMR tubes.

**Scheme 3:**
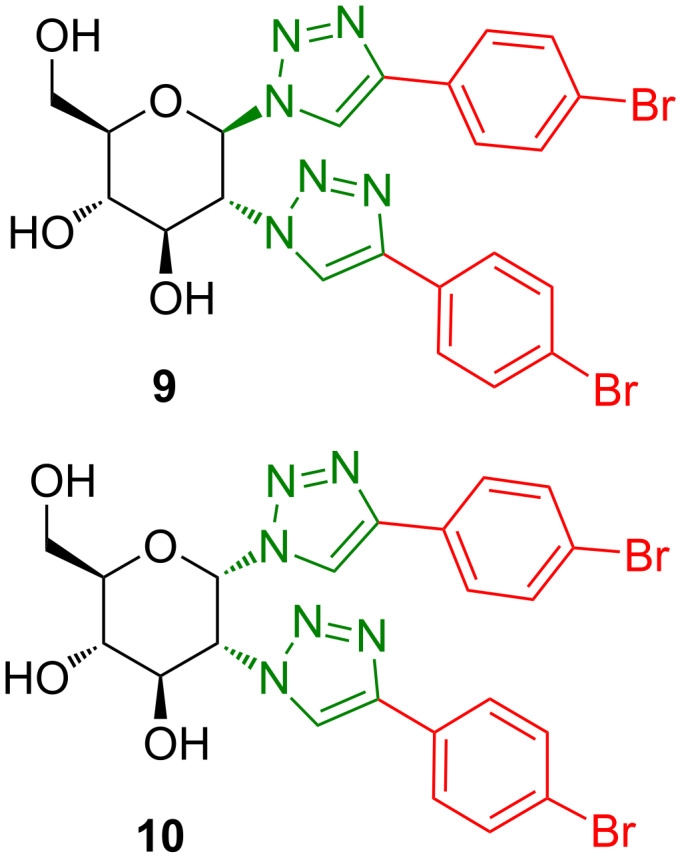
Compounds **9** (*trans*) and **10** (*cis*) [15].

**Scheme 4:**
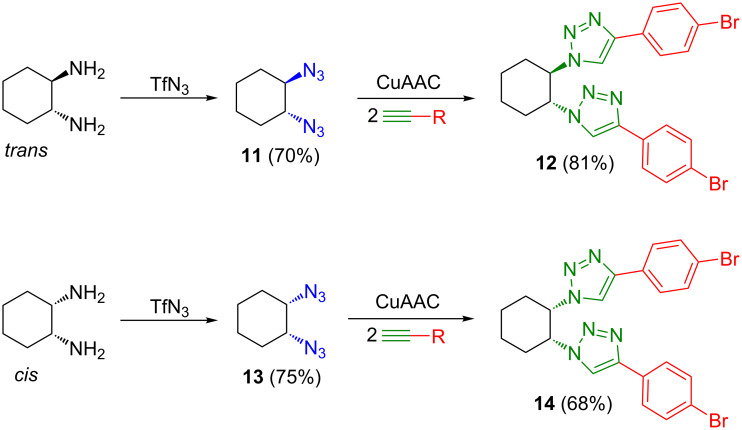
Synthesis of (1*R*,2*R*)- and (1*R*,2*S*)-1,2-bis-(4-(4-bromophenyl)-1*H*-triazol-1-yl)cyclohexane (**12** and **14**).

Three *cis*-/*trans-*configured pairs of compounds were chosen for a gelation ability study. Thus, analogue pairs **8f**/**7f** (Scheme 2), **10**/**9** (Scheme 3), and **14**/**12** (Scheme 4) were selected. The pair **8f**/**7f** was chosen for having different anomeric configurations in the glucosyl system, and therefore different *cis*/*trans* relationships with the equatorial substituent in position 2. Compounds **9** and **10** were selected to monitor the effects of the unprotected hydroxy groups, and the last pair, **14**/**12**, for being a non-glycosyl system, yet with the same *cis*/*trans* relationship as the former compounds. In addition, the available compounds **8a**, **8b**, and **8e** (*cis*) [15] were also tested for gelation to support the results.

Compounds **7** and **8** (Scheme 2) were successfully prepared from the corresponding alkynes and diazides **5** and **6**, respectively. In this context, it was required to isolate **5** and **6** before the coupling reaction could be performed.

Compounds **9** and **10** (Scheme 3) were obtained in good yields by treatment of **7f** and **8f**, respectively, with sodium methoxide in CH_2_Cl_2_/MeOH.

The *trans*- and *cis*-1,2-di(triazol-1-yl)cyclohexanes **12** [14] and **14** (Scheme 4), respectively, were prepared from 1-bromo-4-ethynylbenzene and their corresponding diazides, **11** and **13**, through CuAAC reactions [16–18].

**Figure 1:**
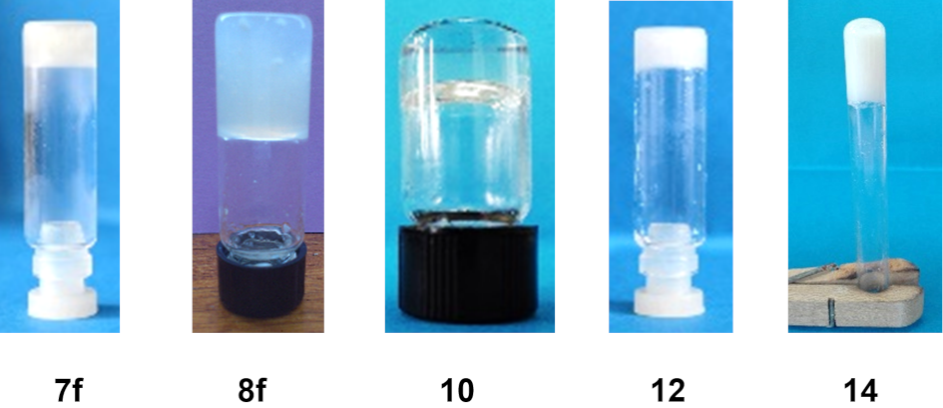
Tube inversion test: gels formed by compounds **7f**, **8f**, **10**, **12**, and **14**.

As can be seen in Figure 1 and Table 1, all these compounds except **9** (having a *trans* configuration) formed gels in DMSO or DMSO/H_2_O mixtures. While the ditriazole species with an α-anomeric configuration, **8f** (*cis*), was able to form gels at different concentrations and DMSO/H_2_O ratios, its β-anomer **7f** (*trans*) only formed a gel when the sample was exposed to a temperature of 8 °C in a refrigerator. This temperature gave rise to an opaque gel that melted above 18 °C (Table 2). The same approach, along with reduction of the minimum gelation concentration to 1.1% w/w (in DMSO) was used to obtain a gel based on **8f**. In addition, this compound proved to be an effective translucent/transparent low-molecular-weight gelator in aqueous solutions of DMSO, forming gels in DMSO/H_2_O, 2:1, v/v at a minimum concentration of 0.8% w/w. The presence of water favored gel formation, decreased the gelation concentration, and the gel's appearance became translucent/transparent. In addition, these gels were stable for several months in sealed vials, although they could be disrupted by agitation or heating. However, the gel state could be restored through cooling.

**Table 1:** Gelation test results for compounds **7f**, **8a**, **8b**, **8e**, **8f**, **9**, **10**, **12**, and **14** (in % w/w).

#	Config	DMSO	DMSO/H_2_O (9:1, v/v)	DMSO/H_2_O (3:1, v/v)	DMSO/H_2_O (2:1, v/v)	DMSO/H_2_O (1:1, v/v)	DMSO/H_2_O (1:2, v/v)

**7f**	β (*trans*)	G 1.1^a^	–	p	p	p	p
**8f**	α (*cis*)	G 5.0 G 1.1^a^	–	G 0.8	G 0.8	p	p
**9**	β (*trans*)	s	s	p	p	p	p
**10**	α (*cis*)	s	–	s	s	G 0.7	G 0.5
**12**	*trans*	G 1.1^a^	p	p	p	p	p
**14**	*cis*	s	G 0.8	p	p	p	p
**8a**	*cis*	–	–	–	G 1.0	susp	susp
**8b**	*cis*	–	–	–	weak G	susp	p
**8e**	*cis*	–	–	–	G 1.0	G 1.0	susp

^a^Gelling at *T* = 8 °C (refrigerator); G = gel; p = precipitate; s = soluble; susp = suspension.

**Table 2:** Critical gelation concentration (CGC, in % w/w) and gel–sol transition temperature (*T*_gs_, via dropping-ball method) for compounds **7f**, **8a**, **8b**, **8e**, **8f**, **10**, **12**, and **14**.

#	Config	Conditions	CGC	*T*_gs_ (°C)	Appearance

**7f**	β (*trans*)	DMSO^a^	1.1	20 ± 2	opaque
**8f**	α (*cis*)	DMSO	5.0	63 ± 1	translucent
**8f**	α (*cis*)	DMSO^a^	1.1	20 ± 2	opaque
**8f**	α (*cis*)	DMSO/H_2_O (3:1, v/v)	0.8	93 ± 3	translucent
**8f**	α (*cis*)	DMSO/H_2_O (2:1, v/v)	0.8	95 ± 3	transparent
**10**	α (*cis*)	DMSO/H_2_O (1:1, v/v)	0.7	68 ± 1	translucent
**10**	α (*cis*)	DMSO/H_2_O (1:2, v/v)	0.5	94 ± 3	transparent
**12**	*trans*	DMSO^a^	1.1	20 ± 2	opaque
**14**	*cis*	DMSO/H_2_O (9:1, v/v)	0.8	50 ± 2^b^	opaque
**8a**	*cis*	DMSO/H_2_O (2:1, v/v)	1.0	87 ± 3	opaque
**8b**	*cis*	DMSO/H_2_O (2:1, v/v)	–	–^c^	–
**8e**	*cis*	DMSO/H_2_O (1:1, v/v)	1.0	96 ± 2	opaque
**8e**	*cis*	DMSO/H_2_O (2:1, v/v)	1.0	92 ± 2	translucent

^a^Gelling at *T* = 8 °C (refrigerator). ^b^Tube inversion test. ^c^Soft gel: dropping-ball method failed.

The triol **9** (*trans*) did not lead to any gel, while its α-anomer **10** (*cis*) produced translucent and transparent gels, respectively, in 1:1 and 1:2 ratios of DMSO/H_2_O, v/v (Table 1 and Table 2). In addition, compound **10** (*cis*) exhibited the lowest minimum gelation concentration, 0.5% w/w in DMSO/H_2_O (1:2, v/v).

Compounds having an α-anomeric configuration (*cis*), independent of the hydroxy groups, were either acetylated (**8f**) or not (**10**), forming gels much easier than their corresponding β-anomers (*trans*). This showed that their ability to form gels was critically dependent on the *cis*/*trans* configuration present in the molecule.

To confirm this result, the supramolecular properties of *trans-* and *cis*-1,2-bis(4-(4-bromophenyl)-1*H*-triazol-1-yl)cyclohexanes **12** and **14**, having the same configuration as compounds **7f** and **8f**, respectively, were analyzed. Both compounds formed gels, but the *trans* stereoisomer **12** did so only at a low temperature (8 °C) in DMSO (G = 1.1, w/w), similar to the β-anomer **7f**, which possessed the same *trans* configuration. On the other hand, the *cis* stereoisomer **14** formed an opaque gel at room temperature in DMSO/H_2_O, 9:1, v/v (G = 0.8, w/w), showing a higher potency for gelation, similar to **8f**. As such, this relationship mirrors the behavior observed for the **7f**/**8f** pair. Table 2 also contains the different *T*_gs_ values obtained by the dropping ball method.

Supportive of these conclusions is the facility of *cis*-configured ditriazoles to form gels. Thus, compound **8a**, with alkyl groups in position 4 on the triazolyl groups, and compound **8e**, with tolyl groups in that position, gelled easily (Table 1 and Table 2).

In addition, 4-methoxycarbonyl-substituted ditriazole **8b** also formed a gel, which was, however, too soft for the dropping ball method to be performed correctly. Therein, the ball dropped down immediately, and changing the DMSO/water ratio did not improve this result. In any case, the results obtained using **8a** and **8e** point at the fact that the presence of either the phenyl groups or the bromine atoms are not strictly necessary for gelation, although both might contribute to an overall stabilization of the supramolecular aggregation.

**Figure 2:**
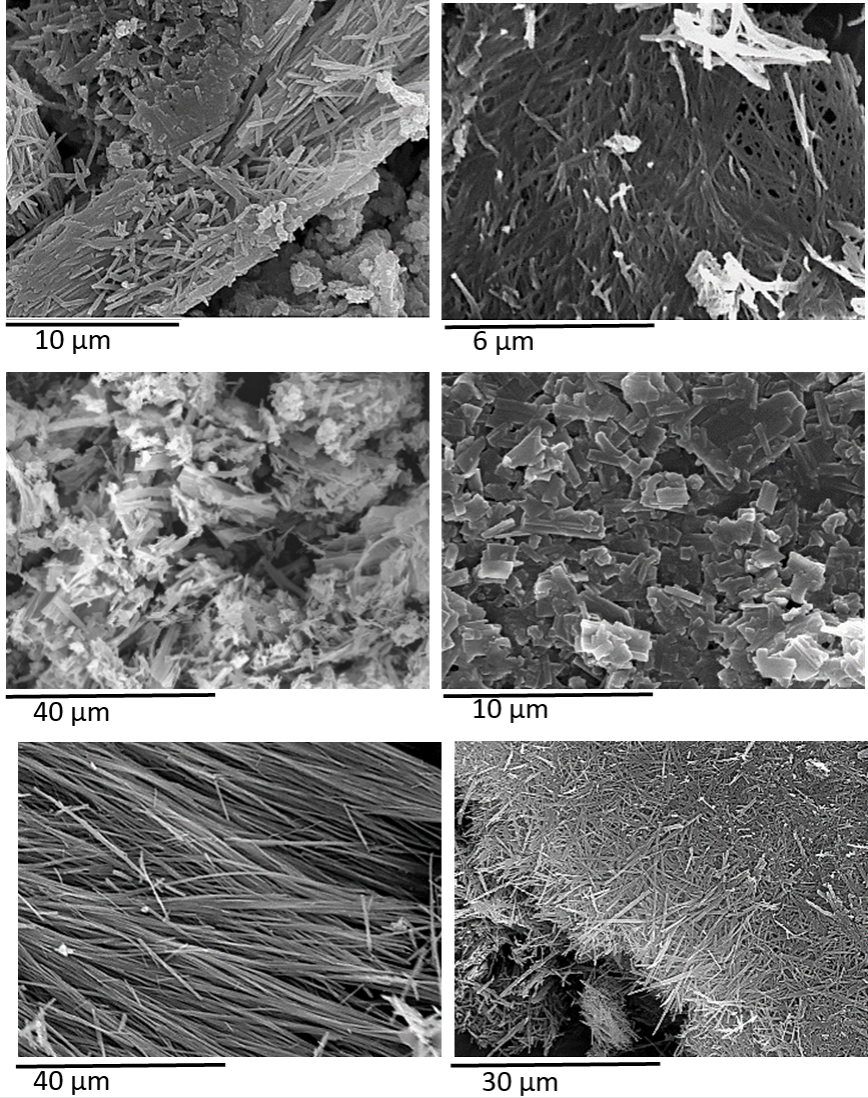
SEM images of the xerogels of compounds **7f** (DMSO, top left), **8f** (DMSO/H_2_O, 3:1, v/v, top right), **10** (DMSO/H_2_O, 1:1, v/v, middle left), **10** (DMSO/H_2_O, 1:2, v/v, middle right), **12** (DMSO, bottom left), and **14** (DMSO/H_2_O, 9:1, v/v, bottom right).

Optical micrographs of the xerogels formed from compounds **7f**, **8f**, **10**, **12**, and **14** were obtained by SEM (Figure 2). Analyses of these images revealed typically fibrous networks for all except **10**. Compound **7f** (*trans*) showed a dense, thin, short, and interlaced/tangled fiber network. The corresponding α-anomer **8f** (*cis*) exhibited an irregular, porous, and dense structure having thin and short fibers. The xerogel of compound **12** showed long, thin, and relatively straight fibers, with lack of torsion, as well as regularity of the network. Its *cis* stereoisomer **14** showed a fibrous network, but of much shorter length. However, the gel of compound **10** showed a different morphology, with short and planar films or scales. This could be due to the high proportion of water in the gel and/or the presence of the three free hydroxy groups in its structure, changing its intermolecular self-assembly behavior.

**Figure 3:**
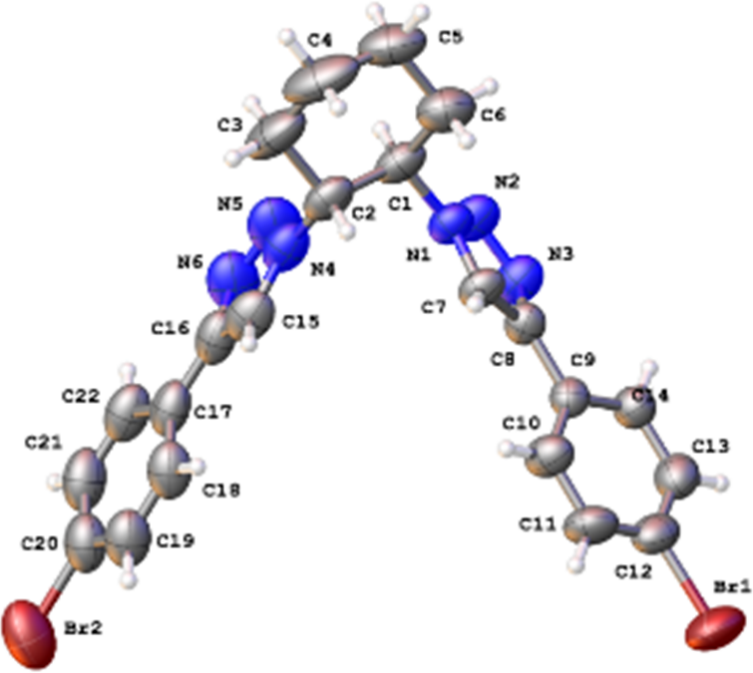
ORTEP representation of the molecular structure of compound **12** (*trans* configuration) obtained from X-ray diffraction data.

**Figure 4:**
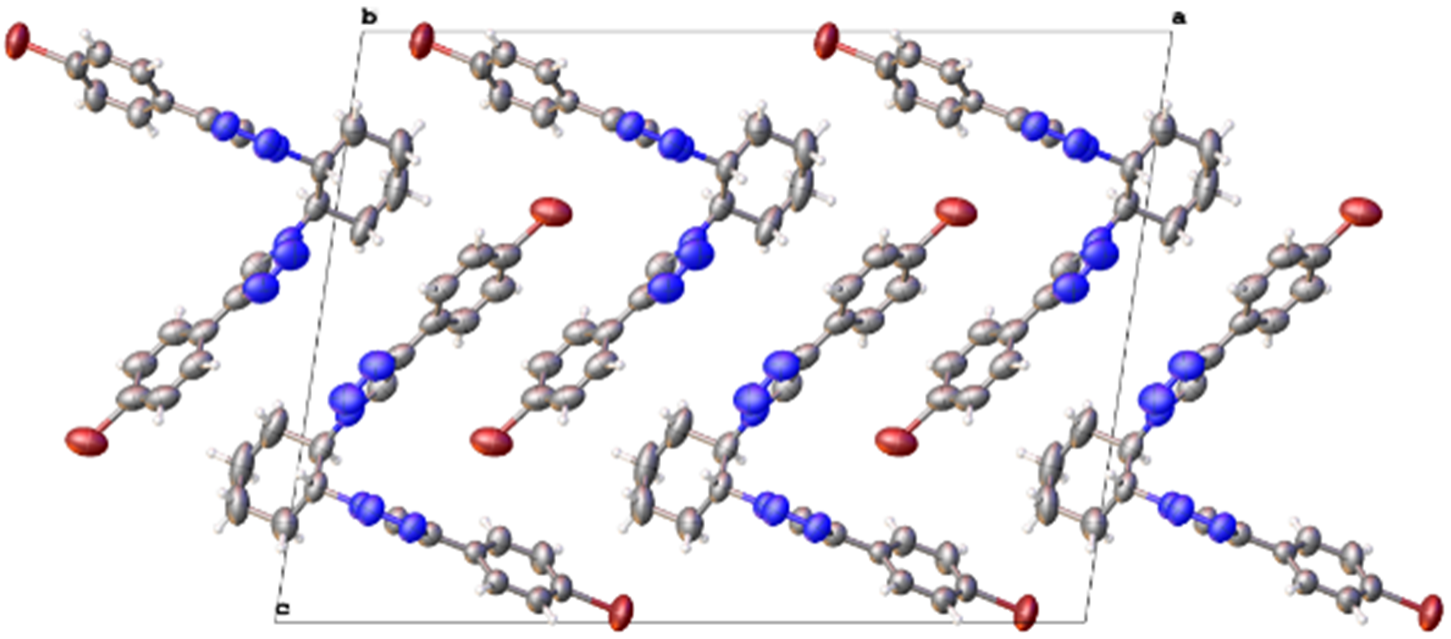
Crystal packing of compound **12** (*trans* configuration) in DMSO.

Various efforts were made to crystallize the obtained compounds; however, only **12** led to single crystals suitable for X-ray crystallography. Its analysis (Figure 3) not only confirmed the structure, but in addition showed a well-organized crystal packing (Figure 4) where the alternate disposition of the molecules displayed a parallelism between the *p*-bromophenyl groups as well as between the triazole rings, and therefore the presence of multiple intermolecular π–π stacking and π–bromine [19] interactions. This compound precipitated in the presence of any amount of water and gelled only in DMSO at a low temperature.

**Figure 5:**
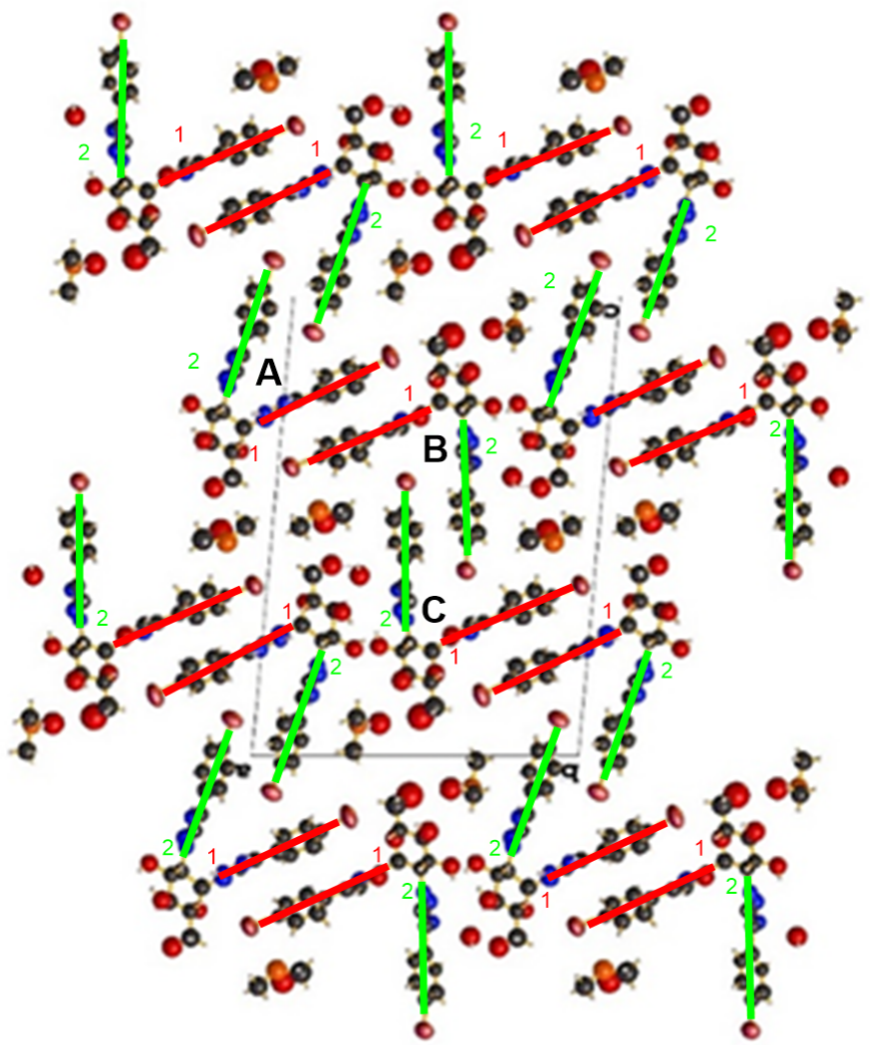
Crystal packing of **10** (*cis* configuration) in DMSO/H_2_O (1:1, v/v). Colored lines: π–π stacking interactions between phenyltriazoles in positions 1 (red lines) and 2 (green lines).

Compound **10** produced pseudo-crystals in DMSO/H_2_O (1:1, v/v) and its X-ray analysis was not accurate enough to resolve its structure. However, its crystal packing (Figure 5) deserves discussion. As with compound **12**, this packing revealed a number of π–bromine interactions [19], together with a high degree of π–π stacking interactions between the aromatic rings, and also that they occured between specific phenyltriazoles. Specifically, these interactions formed between the phenyltriazole moieties in position 1 of a monosaccharide (A) and the phenyltriazole functions in position 1 of its vicinal monosaccharide (B, Figure 5, red lines). Similarly, the phenyltriazole substituents in position 2 of this monosaccharide (B) interacted with a corresponding phenyltriazole in position 2 of the vicinal monosaccharide (C, Figure 5, green lines). In addition, it is worth mentioning the presence of a number of DMSO solvate molecules in the crystal packing next to the hydroxymethyl group in position 6 in the *gg* rotamer, likely linked through hydrogen bonds.

Electronic circular dichroism (ECD) is a powerful technique to study supramolecular systems [20–21], since many interactions responsible for the presence of CD Cotton effects occur through space, such as the well-known ECD exciton chirality method [22–24].

**Figure 6:**
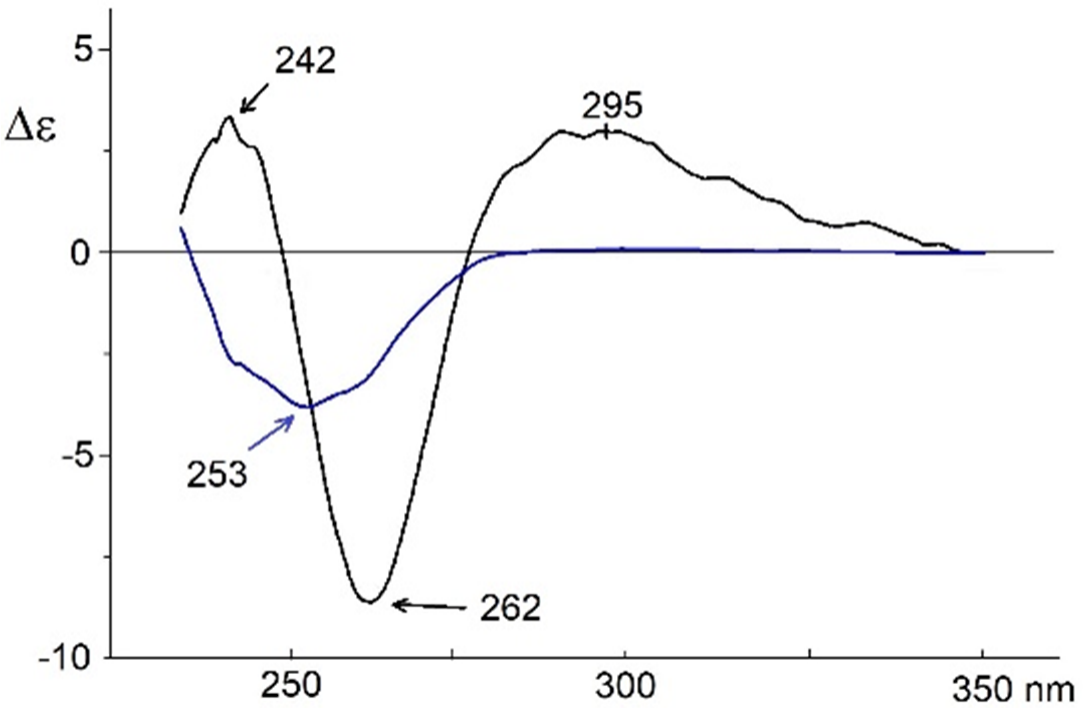
CD spectra of compound **10** (*cis*) in DMSO/H_2_O (1:2, v/v) in solution (in black) and as gel (in blue).

ECD of compound **10** in a DMSO/H_2_O (1:2, v/v) solution and in gel form were successfully measured (Figure 6). ECD of the solution exhibited negative first/positive second exciton Cotton effects at 262 (Δε = −8.6) and 242 nm (Δε = +3.3), respectively (Figure 6, in black), identical to those obtained for this compound in CH_3_CN [15], although of lower intensity. On the other hand, the ECD spectrum of the gel instead exhibited a normal Cotton effect at 253 nm (Δε = −3.8), which, at the same time, was the wavelength of its absorption maximum (Figure 6, in blue). The striking absence of exciton Cotton effects is in complete agreement with the high degree of parallelism between the chromophores, as observed in the crystal packing (Figure 5). Two chromophores can only be coupled under an exciton interaction if the angle between the ^1^L_a_ transitions is different from 0° or 180°, since this interaction is governed by an equation with a vectorial product. The lack of exciton pairwise interactions between the two chromophores in positions 1 and 2 of each molecule could be due to the intercalation of a third chromophore between the two former in a parallel disposition to one of them (see Figure 5).

## Conclusion

In summary, the capacity of di-1,4-disubstituted 1,2,3-triazoles to form gels was studied taking the dependence on the *trans*/*cis* configuration of the molecules into account. The results clearly show that compounds having the *cis* configuration are more prone to forming thermoreversible gels than those with the *trans* configuration. In fact, compound **10**, with an α-anomeric configuration (*cis*), showed the lowest gelation concentration at room temperature (G 0.5% w/w) in DMSO/H_2_O, 1:2, v/v.

The crystal structures obtained for either the *cis* or *trans* configuration through X-ray diffraction studies can be considered good approximations to the supramolecular structure of aryltriazoles in gels. Thus, these compounds showed a high degree of parallelism between the phenyltriazolyl rings, therefore revealing the presence of π–π stacking and π–bromine interactions. In addition, compound **10** (*cis*), with free hydroxy groups, formed hydrogen bonds between the hydroxymethyl groups in position 6 and DMSO molecules while in a *gg* rotamer conformation. The different types of EDC spectra obtained for compound **10** in solution (exciton) and in gel (non-exciton), using DMSO/H_2_O, 1:2, v/v, is in total agreement with the supramolecular structure of the crystal packing of this compound (Figure 5).

## Experimental

### General information

^1^H NMR spectra were recorded at 500 and 600 MHz, and ^13^C NMR spectra at 100, 125, and 150 MHz (VTU 300.0 K). Chemical shifts are reported in ppm. The residual solvent peak was used as an internal reference. HRMS was performed by HRTOFMS in positive mode (ES+). For analytical and preparative thin-layer chromatography, silica gel ready-foils and glass-backed plates (1 mm) were used, respectively. These were visualized using UV light at λ = 254 nm and/or developed by spraying with AcOH/H_2_O/H_2_SO_4_ (20:4:1, v/v/v) and heating to 150 °C. Column chromatography was performed using silica gel (0.015–0.04 mm) and *n*-hexane/EtOAc solvent systems. All reagents were obtained from commercial sources and used without further purification. Solvents were dried and distilled before use. ECD was recorded in the range of 200–400 nm at room temperature by using 10 mm quartz cells. SEM images were taken by a TEM, JEOL JEM 1010 belonging to the Electronic Microscopy Service (SEGAI) of the University of La Laguna, Spain.

Compounds **7f**, **8f**, **9**, **10**, **11**, and **12** were prepared and characterized as described in reference [15].

### Gelation test

Melting points were measured using the dropping ball method. For that, a stainless steel ball of ca. 3 mm diameter (*m* = 130.2 mg) was placed on the gel in a tube with a diameter of 1 cm. This was placed inside an oil bath together with a thermometer, and the temperature of the gel melting was recorded.

### Synthesis and characterization

**1,2-Bis[4-(4-bromophenyl)-1*H*-1,2,3-triazol-1-yl]-1,2-dideoxy-α-ᴅ-glucopyranoside (10):** This compound was prepared as described before [15]. UV (CH_3_CN) λ_max_ (ε) 253 nm (48000); CD (CH_3_CN) λ_ext_ (Δε) 242 (+8.9), 261 (−26.5), 299 nm (+1.1); CD (DMSO:H_2_O, 1:2, v/v) λ_ext_ (Δε) 242 (+3.3), 262 (−8.6), 295 nm (+3.0); CD of gel (DMSO:H_2_O, 1:2, v/v) λ_ext_ (Δε) 253 nm (−3.8).

**(1*R*,2*S*)-1,2-Diazidocyclohexane (13):** For the synthesis of this compound, (1*R*,2*S*)-1,2-diaminocyclohexane and TfN_3_ were required. (a) The triflyl azide was prepared as follows: Sodium azide (922 mg, 14.2 mmol), dissolved in pyridine (15 mL), was cooled to 0 °C under vigorous stirring. Then, triflic anhydride (1.7 mL, 10.1 mmol) was added dropwise, and the reaction mixture was left for 2 h at 0 °C under vigorous stirring. During that time, a small amount of precipitate appeared, which was removed by filtration. The yellow solution was directly used in the next step. (b) To a solution of (1*R*,2*S*)-1,2-diaminocyclohexane (0.42 mL, 3.5 mmol) in 5 mL of pyridine, CuSO_4_·5H_2_O (15 mg, 0.06 mmol) was added while stirring. The mixture was cooled in an ice bath and the above-prepared solution of triflyl azide added dropwise. The resulting green reaction mixture was allowed to warm to room temperature and left for 20 h. The reaction mixture was diluted with CH_2_Cl_2_ (100 mL) and extracted using diluted HCl until the pH value was acidic (4 × 20 mL). The organic phase was washed with NaHCO_3_ (2 × 10 mL) and dried with sodium sulfate. Then, the organic solvent was removed at reduced pressure, keeping the temperature below 30 °C. The residue (437 mg, 75%) showed spectroscopic data according to the literature [25]. ^1^H NMR (600 MHz, CDCl_3_) δ 3.62 (d, *J* = 8.4 Hz, 2H), 1.85 (m, 2H), 1.64 (m, 4H), 1.38 ppm (m, 2H); ^13^C NMR (150 MHz, CDCl_3_) δ 61.4, 27.3, 21.6 ppm.

**(1*R*,2*S*)-1,2-Bis(4-(4-bromophenyl)-1*H*-1,2,3-triazol-1-yl)cyclohexane (14):** To a solution of (1*R*,2*S*)-1,2-diazidocyclohexane (**13**, 363 mg, 2.19 mmol) in H_2_O (22 mL, 22 mmol), 1-bromo-4-ethynylbenzene (913 mg, 5.04 mmol), CuSO_4_·5H_2_O (40 mg, 0.16 mmol) as a 1 M aq. solution, and sodium ascorbate (152 mg 0.77 mmol) as a 1 M aq. solution, were added. The reaction mixture was heated to 70 °C for 48 h. Then, the organic layer was extracted with EtOAc (3 × 100 mL) and the solvent removed at reduced pressure. The residue was chromatographed on silica gel using *n*-hexane/EtOAc to give compound **14** (782 mg, 68% yield). *R*_f_ 0.35 (*n*-hexane/EtOAc, 1:1, v/v); mp 286–288 °C; ^1^H NMR (500 MHz, CDCl_3_) δ 7.46–7.42 (m, 8H), 7.18 (s, 2H), 5.18 (m, 2H), 2.57 (m, 2H), 2.34 (m, 2H), 2.19 (m, 2H), 2.77 ppm (m, 2H); ^13^C NMR (150 MHz, CDCl_3_) δ 146.2, 131.9, 128.9, 127.1, 122.2, 119.9, 60.8, 28.0, 22.3 ppm; HRESIMS (*m*/*z*): [M + Na]^+^ calcd for C_22_H_20_^79^Br_2_N_6_Na, 549.0014; found, 549.0023; [M + Na]^+^ calcd for C_22_H_20_^79^Br^81^BrN_6_Na, 550.9993; found, 551.0006; [M + Na]^+^ calcd for C_22_H_20_^81^Br_2_N_6_Na, 552.9973; found, 552.9977; anal. calcd for C_22_H_20_Br_2_N_6_, C, 50.02; H, 3.82; N, 15.91%; found, C, 49.67; H, 3.90; N, 16.22%.

## Supporting Information

File 1 An SEM image collection of the xerogels and X-ray data for compound **12**.
