# Supplementary material for: Influence of the cis/trans configuration on the supramolecular aggregation of aryltriazoles
Source: Beilstein J Org Chem. 2019 Nov 28;15:2881–8. doi: 10.3762/bjoc.15.282 (PMC6902786; doi:10.3762/bjoc.15.282)
Supplement: File 1 — An SEM image collection of the xerogels and X-ray data for compound 12. [file Beilstein_J_Org_Chem-15-2881-s001.pdf]

BEILSTEIN JOURNAL OF ORGANIC CHEMISTRY

Supporting Information

for

Influence of the *cis*/*trans* configuration on the supramolecular aggregation of aryltriazoles

Sara Tejera, Giada Caniglia, Rosa L. Dorta, Andrea Favero, Javier González-Platas and Jesús T. Vázquez

Beilstein J. Org. Chem. **2019,** *15,* 2881–2888. doi:10.3762/bjoc.15.282

An SEM image collection of the xerogels and X-ray data for compound 12

License and Terms: This is a supporting information file under the terms of the Creative Commons Attribution License (http://creativecommons.org/licenses/by/4.0). Please note that the reuse, redistribution and reproduction in particular requires that the authors and source are credited.
The license is subject to the *Beilstein Journal of Organic Chemistry* terms and conditions: (https://www.beilstein-journals.org/bjoc)

Table of contents:

1. SEM image collection of xerogels S2

2. X-ray data S10

3. References S21

1. SEM image collection of xerogels

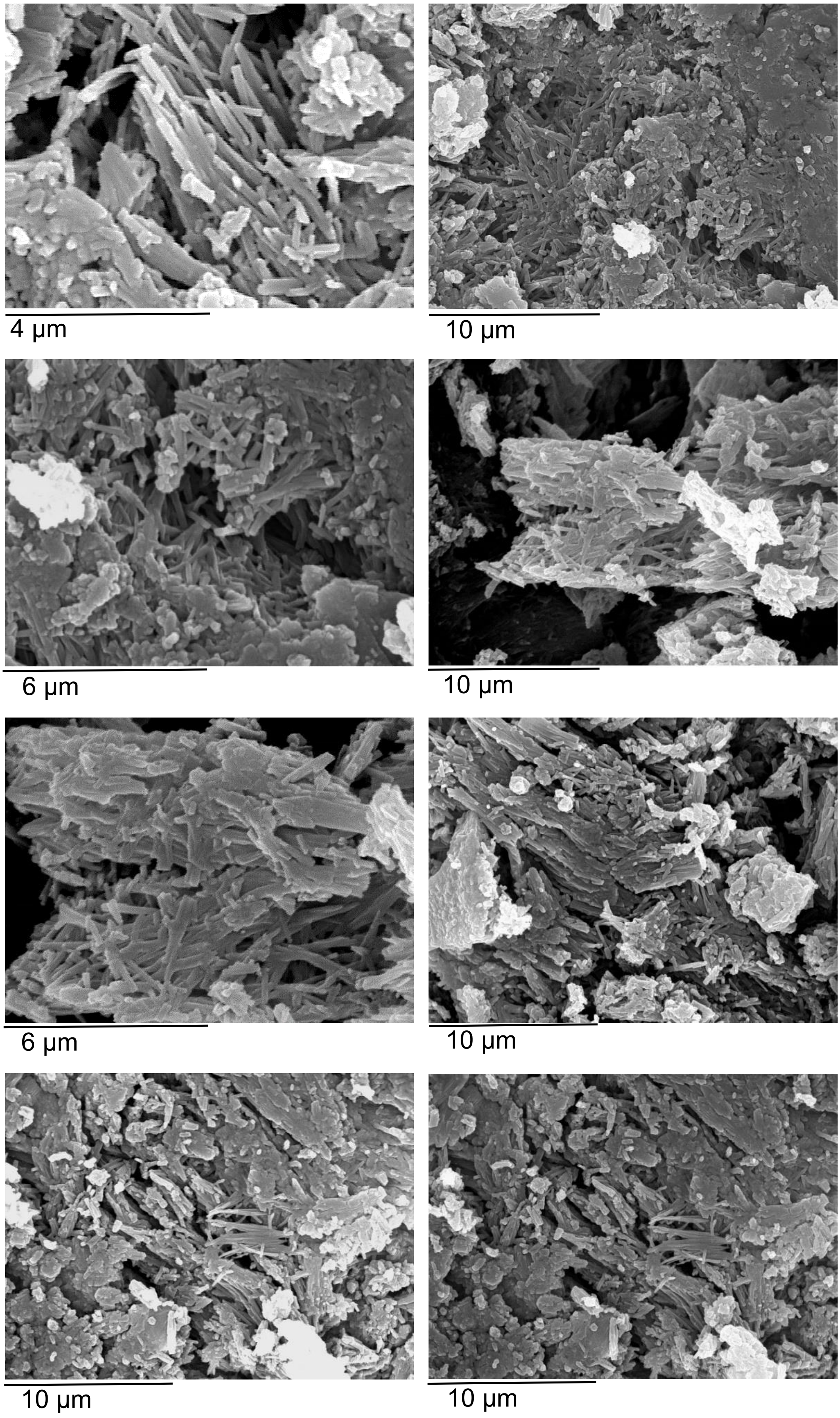

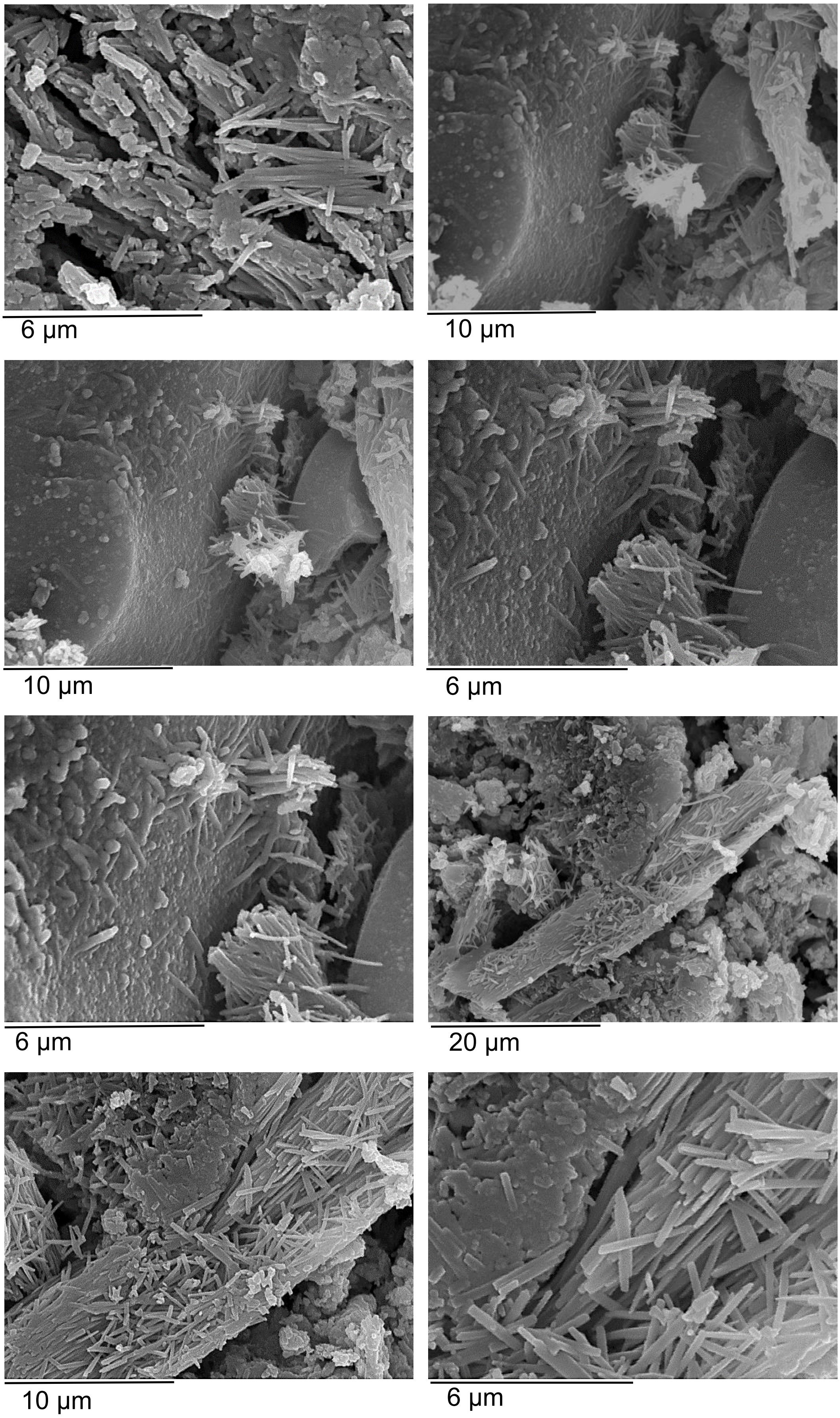

Figure S1. Images of the xerogel **7f** *(trans)*, DMSO, 1.1 % w/w.

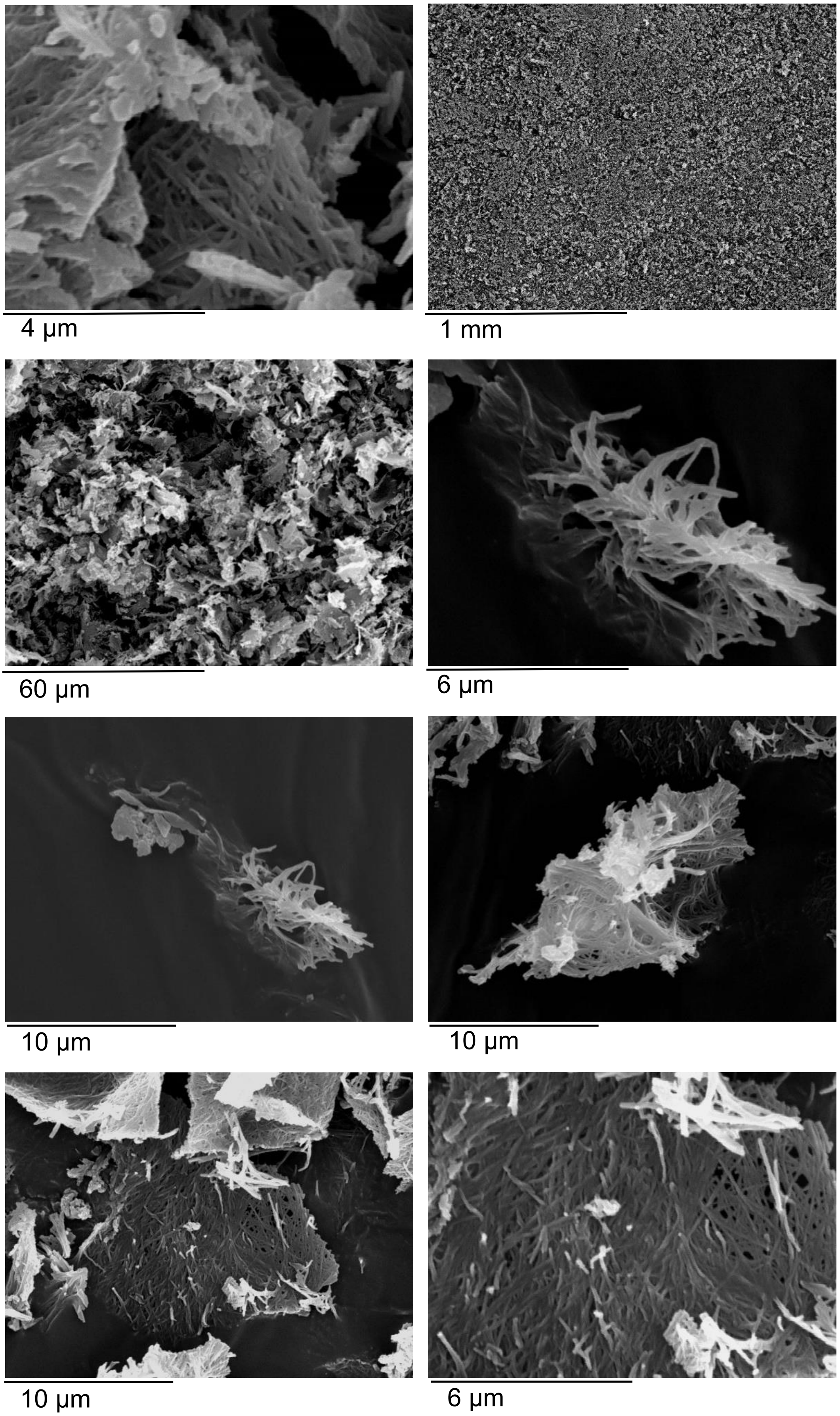

Figure S2. Images of the xerogel **8f** *(cis)*, DMSO/H_2O (3:1), 0.8 % w/w.

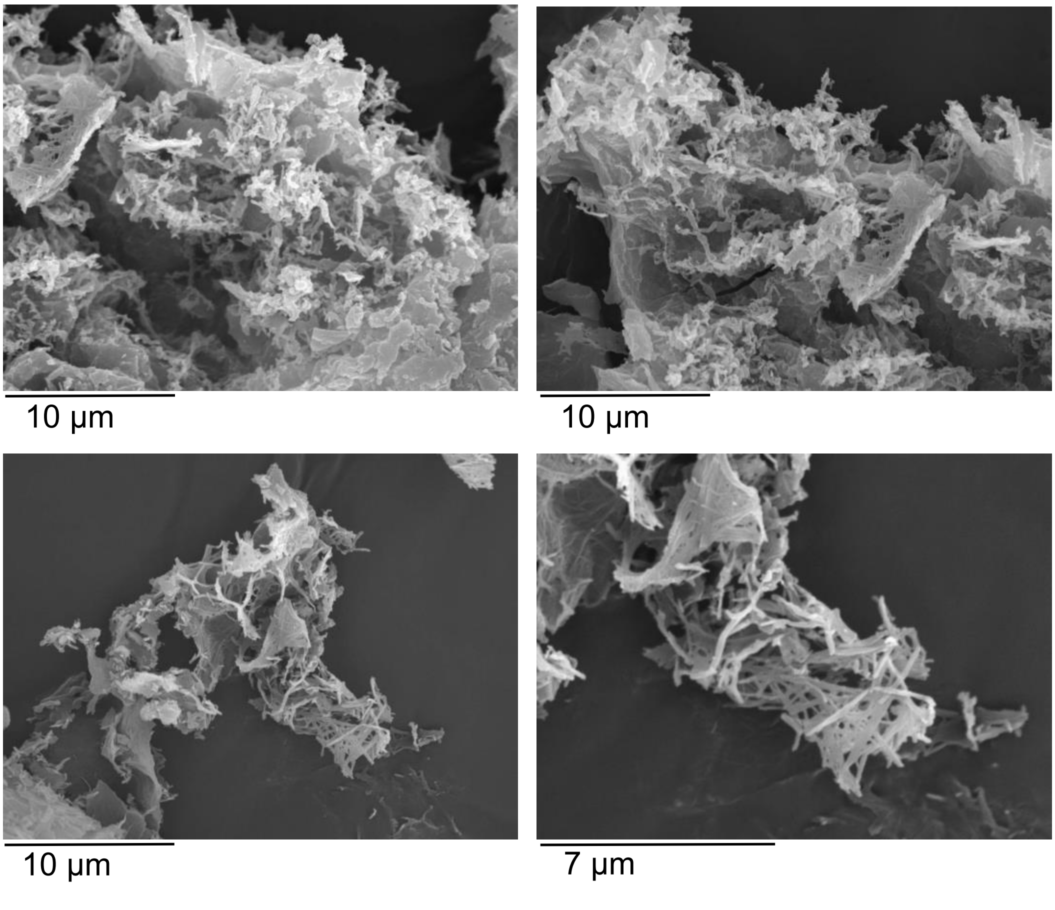

Figure S3. Images of the xerogel **8f** *(cis)*, DMSO/H_2O (2:1), 0.8 % w/w.

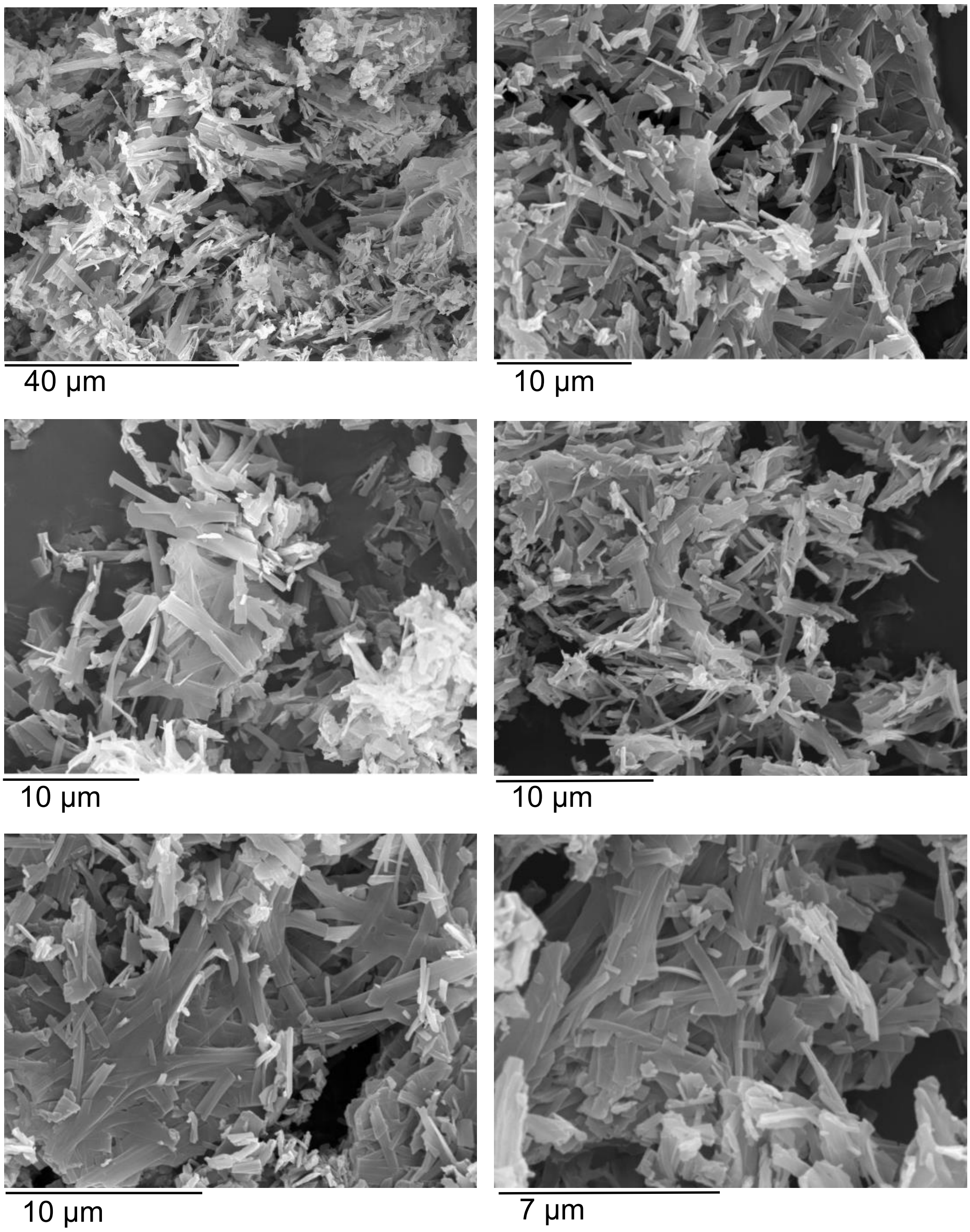

Figure S4. Images of the xerogel **10** *(cis),* DMSO/H_2O (1:1), 0.7 % w/w.

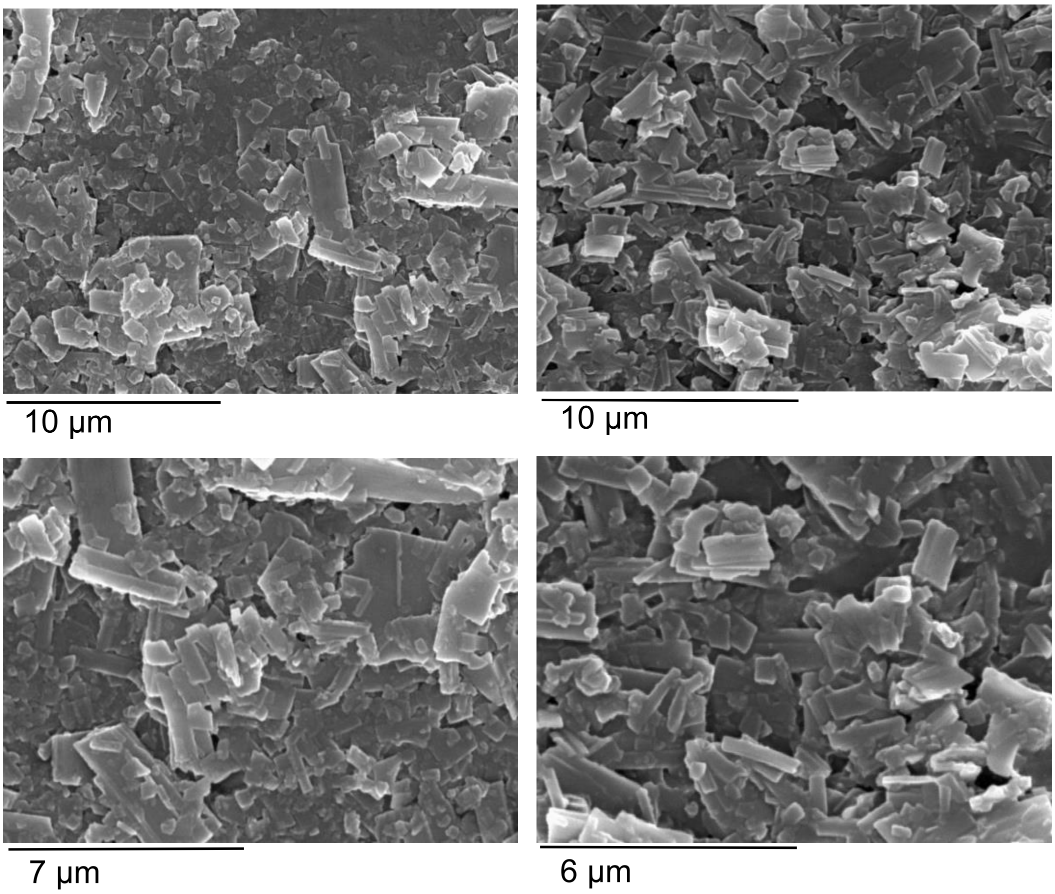

Figure S5. Images of the xerogel **10** *(cis),* DMSO/H_2O (1:2), 0.5 % w/w**.**

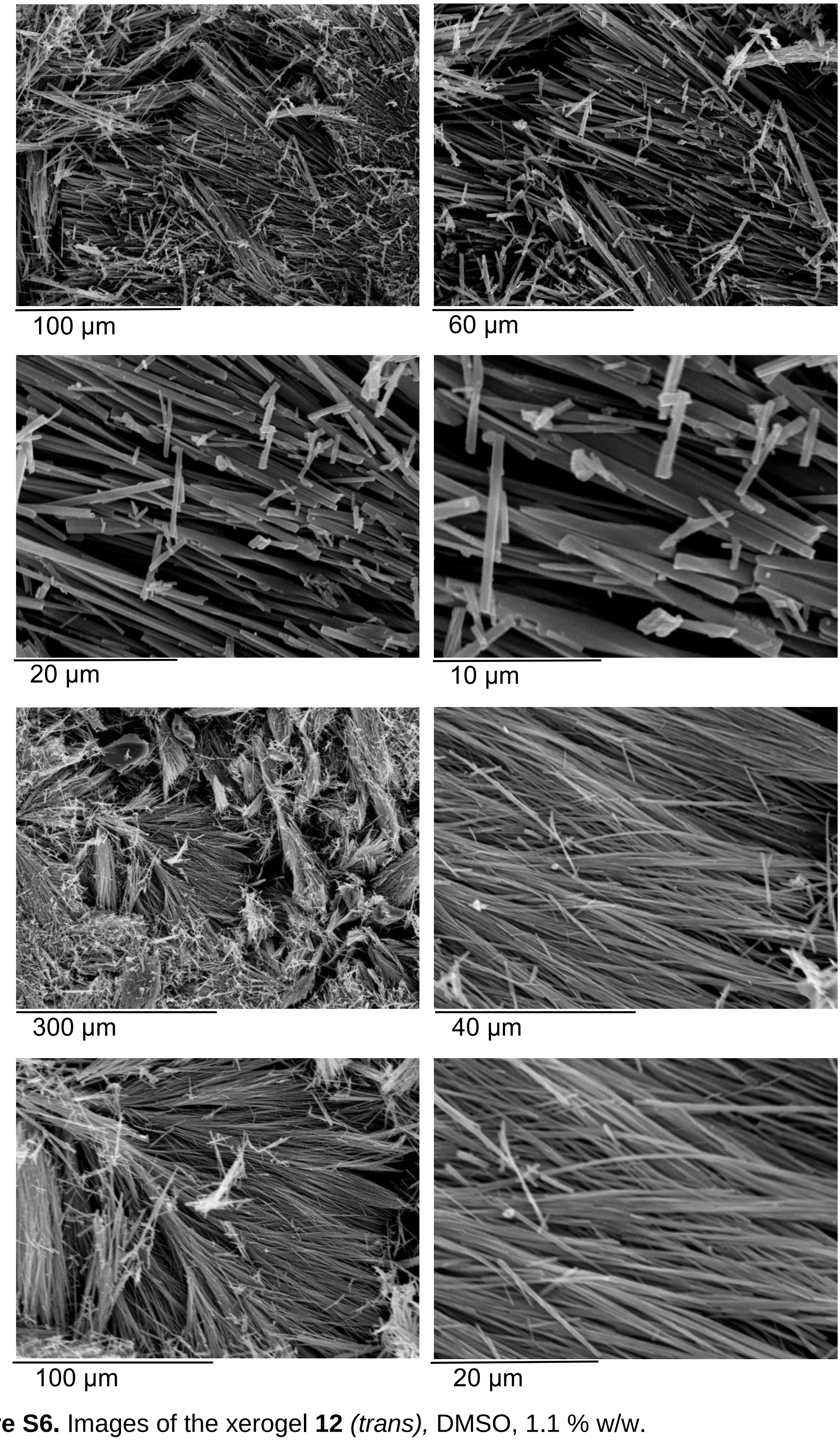

Figure S6. Images of the xerogel **12** *(trans),* DMSO, 1.1 % w/w.

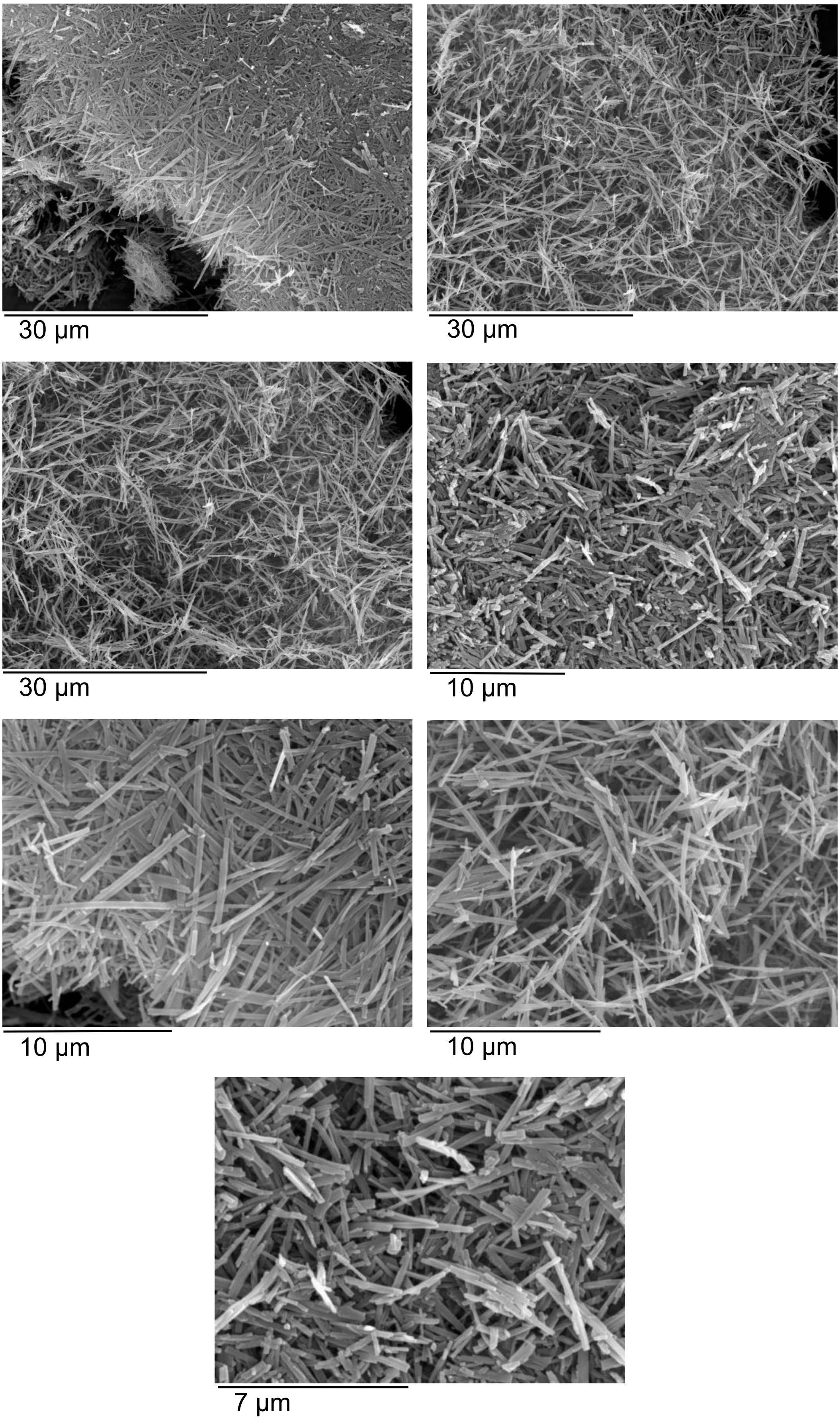

Figure S7. Images of the xerogel **14** *(cis),* DMSO/H_2O (9:1), 0.8 % w/w.

2. X-ray diffraction analysis

Studies of compounds **10** and **12** were carried out by J. González-Platas, X-ray Diffraction Service (SEGAI) of the University of La Laguna, Spain. Crystallographic data for compound **12** have been deposited with the Cambridge Crystallographic Data Centre as supplementary publication numbers CCDC 1941278. Copies of the data can be obtained, free of charge, on application to CCDC, 12 Union Road, Cambridge CB2 1EZ, UK [fax: +44(0)-1223-336033 or e-mail: deposit@ccdc.cam.ac.uk].

Experimental. Single colorless needle-shaped crystals of compound **12** were obtained by recrystallization from DMSO. A suitable crystal 0.29 × 0.02 × 0.02 mm^3 was selected and mounted on an appropiate support on a SuperNova, Dual, Cu at home/near, Atlas diffractometer. The crystal was kept at a steady T = 298(1) K during data collection. The structure was solved with the ShelXT [1] structure solution program using the Intrinsic Phasing solution method, using Olex2 [2] as the graphical interface. The model was refined with version 2018/3 of ShelXL [3] applying Least Squares minimization.

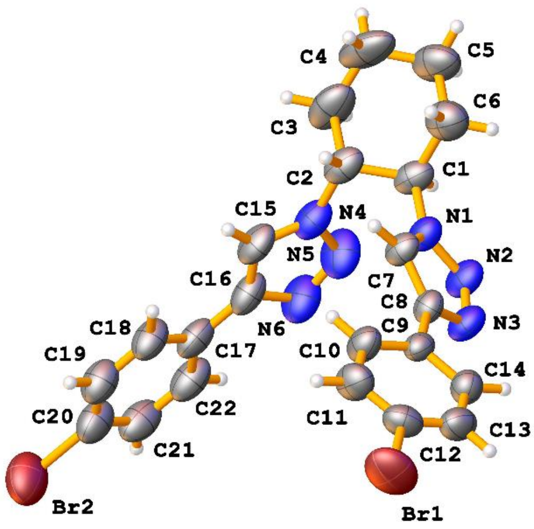

Figure S8. ORTEP representation of the molecular structure of compound **12** (*trans*) from X-ray.

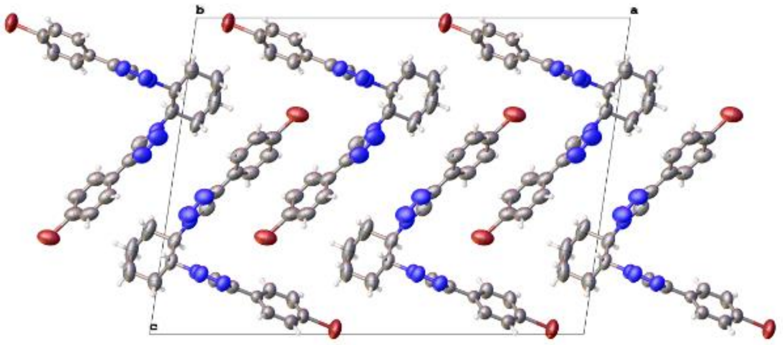

Figure S9. Crystal pack formed by compound **12** (*trans*) in DMSO.

Crystal data. $C_{22}H_{20}Br_2N_6$, M_r = 528.26, monoclinic, $C2$ (No. 5), a = 23.5090(15) Å, b = 5.4892(4) Å, c = 17.3324(13) Å, β = 98.395(7)°, α = γ = 90°, V = 2212.7(3) Å^3, T = 298(1) K, Z = 4, Z' = 1, μ(CuK$_\alpha$) = 4.814, 4325 reflections measured, 2733 unique (R_{int} = 0.0273) which were used in all calculations. The final wR_2 was 0.1076 (all data) and R_1 was 0.0384 (I > 2(I)).

**Compound**	**12**
Formula	$C_{22}H_{20}Br_2N_6$
$D_{calc.}$/ g cm^{-3}	1.586
μ/mm^{-1}	4.814
Formula Weight	528.26
Colour	colourless
Shape	needle
Size/mm^3	0.29×0.02×0.02
T/K	298(1)
Crystal System	monoclinic
Flack Parameter	0.01(3)
Hooft Parameter	-0.01(2)
Space Group	*C*2
a/Å	23.5090(15)
b/Å	5.4892(4)
c/Å	17.3324(13)
α/°	90
β/°	98.395(7)
γ/°	90
V/Å^3	2212.7(3)
Z	4
Z'	1
Wavelength/Å	1.54184
Radiation type	CuK$_\alpha$
Θ_{min}/°	3.801

$\Theta_{max}/^{\circ}$	65.064
Measured Refl.	4325
Independent Refl.	2733
Reflections with I > 2(I)	2383
R_{int}	0.0273
Parameters	271
Restraints	1
Largest Peak	0.321
Deepest Hole	-0.615
GooF	1.039
wR_2 (all data)	0.1076
wR_2	0.1027
R_1 (all data)	0.0442
R_1	0.0384

A colorless needle-shaped crystal with dimensions 0.29 × 0.02 × 0.02 mm^3 was mounted on a suitable support. Data were collected in a SuperNova, Dual, Cu at home/near, Atlas diffractometer operating at T = 298(1) K.

Data were measured using ω scans with CuK_{α} radiation. The total number of runs and images was based on the strategy calculation from the program CrysAlisPro [4]. The maximum resolution that was achieved was $\Theta = 65.064^{\circ}$ (0.85 Å).

The diffraction pattern was indexed. The total number of runs and images was based on the strategy calculation from the program CrysAlisPro [4] and the unit cell was refined using CrysAlisPro on 1889 reflections, 44% of the observed reflections.

Data reduction, scaling and absorption corrections were performed using CrysAlisPro [4].

The final completeness is 99.60% out to 65.064° in Θ. A Gaussian absorption correction was performed using CrysAlisPro 1.171.40.53. Numerical absorption correction based on gaussian integration over a multifaceted crystal model Empirical absorption correction using spherical harmonicas implemented in SCALE3 ABSPACK. The absorption coefficient μ of this material is 4.814 mm^{-1} at this wavelength (λ = 1.542 Å) and the minimum and maximum transmissions are 0.546 and 1.000.

The structure was solved and the space group *C*2 (# 5) determined by the ShelXT [1] structure solution program using Intrinsic Phasing and refined by Least Squares using version 2018/3 of ShelXL [3]. All non-hydrogen atoms were refined anisotropically. Hydrogen atom positions were calculated geometrically and refined using the riding model. Hydrogen atom positions were calculated geometrically and refined using the riding model.

Table S1: Fractional atomic coordinates (×10^4) and equivalent isotropic displacement parameters (Å^2 × 10^3) for compound **12**. U_{eq} is defined as 1/3 of the trace of the orthogonalised U_{ij}.

Atom	x	y	z	U_{eq}
Br1	750.4(2)	4055(3)	161.8(5)	111.8(4)
Br2	2340.9(5)	3805(2)	6944.5(4)	113.3(3)
N2	4028(2)	8231(8)	1901(3)	64.3(12)
N1	4160.0(18)	5864(8)	1981(3)	54.0(10)
N3	3479.2(19)	8389(8)	1604(3)	62.9(12)
N4	4461.2(19)	4283(9)	3559(3)	64.9(12)
C9	2657(2)	5625(9)	1183(3)	46.5(10)
C7	3697.7(18)	4473(9)	1727(3)	52.5(11)
C8	3261(2)	6103(9)	1491(3)	49.1(11)

Atom	x	y	z	U_{eq}
N6	4214(3)	6989(10)	4343(4)	85.9(17)
N5	4523(3)	6642(11)	3778(4)	87.6(18)
C10	2392(2)	3547(12)	1375(3)	63.8(13)
C12	1530(2)	4727(13)	583(3)	65.7(16)
C17	3576(3)	4625(10)	5085(3)	65.9(15)
C13	1784(3)	6801(13)	382(3)	66.4(15)
C11	1823(2)	3079(12)	1087(4)	72.4(17)
C16	3958(3)	4842(10)	4491(3)	66.5(15)
C2	4753(2)	3302(10)	2954(3)	64.1(14)
C14	2351(2)	7276(11)	680(3)	57.4(13)
C15	4112(3)	3158(10)	3991(4)	68.6(15)
C21	3191(3)	6193(13)	6205(4)	81(2)
C22	3554(3)	6436(12)	5647(4)	76.0(18)
C1	4750(2)	5118(11)	2292(4)	61.7(14)
C20	2844(3)	4146(15)	6198(3)	78.0(16)
C18	3235(3)	2600(11)	5099(4)	71.2(17)
C19	2863(3)	2367(13)	5649(4)	77.2(18)
C3	5372(3)	2553(16)	3286(5)	99(3)
C4	5699(3)	1648(17)	2648(7)	115(3)
C5	5683(3)	3475(16)	1996(5)	96(2)
C6	5063(2)	4144(18)	1658(4)	84.7(17)

Table S2: Anisotropic displacement parameters (×10^4) compound **12**. The anisotropic displacement factor exponent takes the form: $-2\pi^2[h^2a^{*2} \times U_{11} + ... +2hka^* \times b^* \times U_{12}]$

Atom	U_{11}	U_{22}	U_{33}	U_{23}	U_{13}	U_{12}
Br1	51.7(3)	159.8(9)	120.2(5)	-4.3(8)	-0.1(3)	-15.9(5)
Br2	167.4(8)	98.4(6)	77.2(4)	-1.9(5)	28.3(4)	-3.6(8)
N2	66(3)	36(2)	87(3)	-1(2)	-2(2)	-8(2)
N1	53(2)	39(2)	68(2)	2(2)	-0.6(19)	-5.7(19)
N3	67(3)	37(3)	81(3)	0(2)	-2(2)	1(2)
N4	71(2)	39(2)	77(3)	-2(2)	-11(2)	-8(2)
C9	51(2)	40(3)	49(2)	0(2)	7.9(19)	6(2)
C7	49(2)	34(3)	71(3)	-1(2)	0(2)	-6(2)
C8	57(3)	34(2)	54(2)	2(2)	3(2)	-5(2)
N6	121(5)	48(3)	85(3)	-7(3)	4(3)	-17(3)
N5	120(5)	51(3)	89(4)	-11(3)	8(4)	-29(3)
C10	57(3)	49(3)	84(3)	11(3)	3(2)	-3(3)
C12	48(2)	79(5)	71(3)	-10(3)	10(2)	2(3)
C17	84(3)	43(3)	63(3)	3(2)	-16(3)	5(3)
C13	60(3)	73(4)	66(3)	5(3)	7(3)	12(3)
C11	55(3)	64(4)	100(4)	3(3)	14(3)	-12(3)
C16	86(4)	40(3)	65(3)	5(3)	-16(3)	-6(3)
C2	55(3)	41(3)	90(3)	-3(3)	-10(3)	-7(2)
C14	59(3)	53(3)	60(3)	5(3)	8(2)	-1(3)
C15	78(3)	35(3)	86(4)	4(3)	-9(3)	-8(3)
C21	114(5)	58(4)	63(3)	-10(3)	-9(4)	13(4)
C22	95(4)	45(3)	78(4)	-2(3)	-19(4)	-3(3)
C1	46(2)	45(3)	90(4)	-5(3)	-6(2)	-4(2)

Atom	U_{11}	U_{22}	U_{33}	U_{23}	U_{13}	U_{12}
C20	107(4)	60(4)	62(3)	4(3)	-4(3)	6(4)
C18	104(5)	43(3)	63(3)	-2(3)	-1(3)	1(3)
C19	105(5)	52(3)	70(3)	0(3)	-4(4)	-5(3)
C3	59(3)	81(5)	143(6)	23(5)	-25(4)	-7(4)
C4	51(4)	74(5)	212(10)	5(7)	-7(5)	6(4)
C5	56(3)	82(5)	153(6)	-2(6)	20(3)	-4(4)
C6	67(3)	78(4)	110(4)	-5(5)	14(3)	-10(4)

Table S3: Bond Lengths in Å for compound **12**.

Atom	Atom	Length/Å	Atom	Atom	Length/Å
Br1	C12	1.907(5)	N6	C16	1.365(9)
Br2	C20	1.886(7)	C10	C11	1.382(8)
N2	N1	1.338(7)	C12	C13	1.355(9)
N2	N3	1.321(6)	C12	C11	1.371(9)
N1	C7	1.349(6)	C17	C16	1.467(9)
N1	C1	1.470(7)	C17	C22	1.398(9)
N3	C8	1.359(7)	C17	C18	1.372(9)
N4	N5	1.351(8)	C13	C14	1.381(8)
N4	C2	1.439(8)	C16	C15	1.352(9)
N4	C15	1.340(8)	C2	C1	1.519(8)
C9	C8	1.466(7)	C2	C3	1.542(8)
C9	C10	1.364(8)	C21	C22	1.385(11)
C9	C14	1.386(7)	C21	C20	1.388(11)
C7	C8	1.377(7)	C1	C6	1.508(9)
N6	N5	1.314(9)	C20	C19	1.368(9)

Atom	Atom	Length/Å	Atom	Atom	Length/Å
C18	C19	1.391(10)	C4	C5	1.508(12)
C3	C4	1.519(13)	C5	C6	1.535(9)

Table S4: Bond angles in ° for compound **12**.

Atom	Atom	Atom	Angle/°	Atom	Atom	Atom	Angle/°
N3	N2	N1	107.6(4)	C11	C12	Br1	118.8(5)
N2	N1	C7	110.6(4)	C22	C17	C16	121.4(6)
N2	N1	C1	120.0(4)	C18	C17	C16	119.8(6)
C7	N1	C1	129.3(5)	C18	C17	C22	118.9(7)
N2	N3	C8	108.8(4)	C12	C13	C14	120.0(6)
N5	N4	C2	121.3(5)	C12	C11	C10	118.5(6)
C15	N4	N5	109.5(5)	N6	C16	C17	122.3(6)
C15	N4	C2	129.1(5)	C15	C16	N6	107.6(6)
C10	C9	C8	121.0(5)	C15	C16	C17	130.0(5)
C10	C9	C14	119.0(5)	N4	C2	C1	110.8(5)
C14	C9	C8	120.0(5)	N4	C2	C3	110.6(5)
N1	C7	C8	105.0(5)	C1	C2	C3	111.0(5)
N3	C8	C9	122.9(5)	C13	C14	C9	119.8(5)
N3	C8	C7	108.0(4)	N4	C15	C16	106.6(5)
C7	C8	C9	129.2(5)	C22	C21	C20	119.5(6)
N5	N6	C16	108.8(6)	C21	C22	C17	120.5(7)
N6	N5	N4	107.4(6)	N1	C1	C2	111.1(5)
C9	C10	C11	121.4(5)	N1	C1	C6	111.5(5)
C13	C12	Br1	119.9(5)	C6	C1	C2	111.6(6)
C13	C12	C11	121.3(5)	C21	C20	Br2	120.1(5)

Atom	Atom	Atom	Angle/°
C19	C20	Br2	119.6(6)
C19	C20	C21	120.3(6)
C17	C18	C19	120.9(6)
C20	C19	C18	119.9(7)
C4	C3	C2	111.4(6)
C5	C4	C3	111.7(7)
C4	C5	C6	111.3(6)
C1	C6	C5	110.2(6)

Table S5: Hydrogen fractional atomic coordinates (×10[4]) and equivalent isotropic displacement parameters (Å^2 × 10^3) for compound **12**. U_{eq} is defined as 1/3 of the trace of the orthogonalised U_{ij}.

Atom	x	y	z	U_{eq}
H7	3677.73	2780.72	1714.05	63
H10	2597.85	2422.3	1706.05	77
H13	1577.05	7904.4	43.35	80
H11	1642.44	1678.29	1232.02	87
H2	4545.52	1840.89	2745.35	77
H14	2525.81	8699.29	542.24	69
H15	3998.01	1535.03	3954.16	82
H21	3180.91	7392.59	6581.18	97
H22	3783.93	7813.26	5646.35	91
H1	4955.77	6574.59	2507.13	74
H18	3253.44	1366.79	4735.12	85
H19	2626.95	1004.93	5643.25	93
H3A	5572.4	3939.76	3545.57	118
H3B	5361.38	1276.13	3670.98	118
H4A	6095.71	1334.54	2868.82	138
H4B	5531.58	126.35	2440.84	138
H5A	5876.14	2798.43	1585.88	116
H5B	5888.7	4934.37	2190.12	116
H6A	5065	5364.47	1253.68	102
H6B	4865.33	2712.76	1424.81	102

3. References included in this Supporting Inofmation

1. Sheldrick, G. M. *Acta Cryst.*, **2015**, *A71*, 3–8.

2. Dolomanov O. V.; Bourhis, L. J.; Gildea, R. J.; Howard, J. A. K.; Puschmann, H. *J. Appl. Cryst.*, **2009**, *42*, 339–341.

3. Sheldrick, G. M. *Acta Cryst.*, **2015**, C27, 3–8.

4. CrysAlisPro Software System, Rigaku Oxford Diffraction (2019).
